# Investigating the Role of Obesity, Vitamin D Deficiency, and Eosinophilia in Pediatric Asthma Severity: A Cross-sectional Study

**DOI:** 10.1016/j.opresp.2025.100505

**Published:** 2025-10-15

**Authors:** Mobina Rabiei, Maryam Mohammadi, Saeed Amini, Javad Nazari

**Affiliations:** a Department of Pediatrics, School of Medicine, Arak University of Medical Sciences, Arak, Iran; b Department of Health and Management Sciences, Khomein University of Medical Sciences, Khomein, Iran

**Keywords:** Asthma, Vitamin D deficiency, Eosinophilia, Obesity, Children, Asma, Deficiencia de vitamina D, Eosinofilia, Obesidad, Niños, Adolescentes

## Abstract

**Introduction:**

Modifiable factors such as obesity, vitamin D deficiency, and eosinophilia may contribute to increased asthma severity, but their combined impact remains underexplored, especially in resource-limited settings. This study aimed to investigate the independent and combined associations of obesity, vitamin D deficiency, and eosinophilia with asthma severity in children aged 6–18 years in Arak, Iran.

**Material and methods:**

In this cross-sectional study, 177 children with physician-diagnosed asthma were recruited from the Amir-Kabir Hospital in 2024. Data on body mass index, serum 25-hydroxy vitamin D levels, and eosinophil counts were collected. Asthma severity was categorized as mild, moderate, or severe according to clinical guidelines. Statistical analyses included chi-square tests, ANOVA, and multivariate logistic regression adjusting for age, sex, and family history.

**Results:**

The mean age of participants was 10.4 ± 3.1 years, and 69.5% were male. Overweight/obesity was observed in 30.5%, vitamin D deficiency in 56.5%, and eosinophilia in 10.2% of the children. Asthma severity was mild in 74.6%, moderate in 15.3%, and severe in 10.2% of cases. All three risk factors were significantly more prevalent in children with moderate–severe asthma (*p* < 0.001). Multivariate analysis revealed that overweight/obesity (adjusted overall risk (OR): 3.21; 95% confidence interval (95% CI): 1.87–5.51), vitamin D deficiency (adjusted OR: 2.76; 95% CI: 1.45–5.25), and eosinophilia (adjusted OR: 8.42; 95% CI: 3.24–21.89) were independently associated with increased asthma severity.

**Conclusion:**

Obesity, vitamin D deficiency, and eosinophilia are independently associated with greater asthma severity in children. Addressing these risk factors through integrated clinical assessments and public health interventions may improve asthma outcomes.

## Graphical abstract

**Figure:**
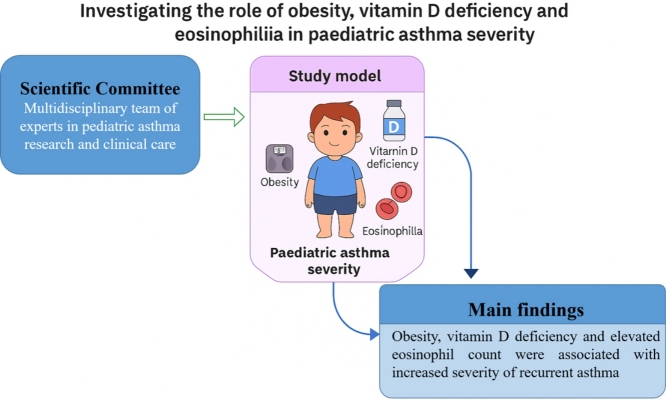

## Abstract (es)

**Introducción:**

Determinados factores modificables, como la obesidad, la deficiencia de vitamina D y la eosinofilia, pueden contribuir a agravar el asma, pero su efecto conjunto no se ha estudiado lo suficiente, sobre todo en entornos de recursos limitados. El objetivo de este estudio ha sido investigar las asociaciones independientes y combinadas de la obesidad, la deficiencia de vitamina D y la eosinofilia con la gravedad del asma en niños y adolescentes de 6 a 18 años en la ciudad de Arak (Irán).

**Material y métodos:**

Se realizó un estudio transversal en 2024, en el Hospital Amir Kabir de la ciudad iraní de Arak, en el que participaron 177 menores afectos de asma con diagnóstico facultativo. Se recopilaron datos sobre el índice de masa corporal, los niveles séricos de 25-hidroxivitamina D y las cifras de eosinófilos. Se clasificó el asma según fuera leve, moderada o grave, con arreglo a las directrices clínicas. Como análisis estadísticos se efectuaron pruebas de la χ^2^, ANOVA y regresiones logísticas multivariante con ajustes por edad, sexo y antecedentes familiares.

**Resultados:**

La media de edad de los participantes era de 10,4 ± 3,1 años, y el 69,5% eran varones. El 30,5% presentaban obesidad o sobrepeso; el 56,5%, deficiencia de vitamina D, y el 10,2%, eosinofilia. El asma era leve en el 74,6% de los casos, moderada en el 15,3% y grave en el 10,2%. Los 3 factores de riesgo eran significativamente más prevalentes en los pacientes con asma moderada o grave (p < 0,001). Los análisis multivariantes revelan una asociación independiente entre una mayor gravedad del asma y el sobrepeso u obesidad (riesgo general [RG] ajustado: 3,21; intervalo de confianza [IC] del 95%: 1,87-5,51), la deficiencia de vitamina D (RG ajustado: 2,76; IC 95%: 1,45-5,25) y la eosinofilia (RG ajustado: 8,42; IC 95%: 3,24-21,89).

**Conclusión:**

La obesidad, la deficiencia de vitamina D y la eosinofilia guardan una asociación independiente con una mayor gravedad del asma en los niños y adolescentes. Abordando estos factores de riesgo con evaluaciones clínicas integradas e intervenciones de salud pública, se podría mejorar la morbilidad por asma.

## Introduction

Asthma is one of the most prevalent chronic respiratory diseases affecting children worldwide. This has significant public health implications due to its impact on morbidity, healthcare utilization, and quality of life.^1^, ^2^ The World Health Organization and the Global Initiative for Asthma (GINA) report that asthma affects over 300 million people globally, with a particularly high prevalence among children in both developed and developing countries.^3^ The rising incidence of childhood asthma has been attributed to a complex interplay of genetic, environmental, and lifestyle factors, making it a priority for public health interventions and research.^4^

In children, asthma typically manifests as recurrent episodes of wheezing, dyspnea, chest tightness, and cough, which can disrupt daily activities, impair school performance, and increase the risk of hospitalization.^5^ The burden of asthma is especially pronounced in low- and middle-income countries, where limited access to healthcare and poor disease management can exacerbate outcomes.^6^ Despite advances in medical therapy, a substantial proportion of children continue to experience uncontrolled asthma, highlighting the need for a deeper understanding of modifiable risk factors that contribute to disease severity.^7^

Recent epidemiological studies have identified several potentially modifiable risk factors for asthma severity, including obesity, eosinophilic inflammation, and vitamin D deficiency.^8^ Obesity, in particular, has emerged as a significant concern, with a growing body of evidence linking excess body weight to increased asthma incidence, more severe symptoms, and reduced response to standard therapies.^9^ The mechanisms underlying this association are thought to involve systemic inflammation, altered lung mechanics, and changes in immune function, though the precise pathways remain an area of active investigation.^10^

Vitamin D deficiency is another factor increasingly recognized for its role in asthma pathogenesis and severity.^11^ Vitamin D is known to modulate immune responses and reduce airway inflammation, and low levels have been associated with impaired lung function, increased airway hyperresponsiveness, and reduced efficacy of corticosteroid therapy in children with asthma.^12^ Observational studies suggest that vitamin D supplementation may improve asthma control, although randomized controlled trials have yielded mixed results, underscoring the need for further research in diverse populations.^13^

Eosinophilic inflammation is a hallmark of allergic asthma and is commonly detected in children with severe or poorly controlled disease.^14^ Elevated eosinophil counts are associated with increased airway inflammation, more frequent exacerbations, and greater healthcare utilization, making eosinophilia a valuable biomarker for risk stratification and targeted therapy.^15^ However, the relationship between eosinophilia and other risk factors, such as obesity and vitamin D deficiency, is not fully understood, particularly in pediatric populations from resource-limited settings.^16^

Despite the increasing recognition of these modifiable risk factors, scant data are available on their combined impact on asthma severity in children, especially in regions with high asthma prevalence and limited healthcare resources.^17^ Most existing studies have focused on individual risk factors in isolation, and few have examined the synergistic effects of obesity, vitamin D deficiency, and eosinophilia in the context of childhood asthma.^8^ Addressing this knowledge gap is critical for developing targeted public health strategies that can reduce the burden of asthma and improve outcomes for affected children.

This study aimed to evaluate the associations between obesity, vitamin D deficiency, and eosinophilia with asthma severity in children aged 6–18 years in Arak, Iran. By examining these risk factors in combination, we sought to provide a more comprehensive understanding of their roles in asthma severity and to inform the development of effective, evidence-based interventions in pediatric asthma management.

## Material and methods

### Study design and setting

This cross-sectional study was conducted at Amir-Kabir Hospital, a major referral center in Arak, Iran, from January to December 2024. The study aimed to evaluate the associations between obesity, vitamin D deficiency, eosinophilia, and asthma severity in children aged 6–18 years. Cross-sectional designs are well-suited for assessing prevalence and exploring multiple risk factors simultaneously, as recommended by public health research guidelines.^18^

### Participants

Children aged 6–18 years with a physician-confirmed diagnosis of asthma, based on established clinical criteria and supported by spirometry (when feasible), were eligible for inclusion.^19^ Participants were recruited consecutively from the pediatric respiratory medicine clinic of Amir-Kabir Hospital to minimize selection bias. Exclusion criteria comprised incomplete medical records, inability to obtain blood samples, or the presence of other chronic respiratory diseases (e.g., cystic fibrosis, chronic lung disease). Informed consent was obtained from all participants’ parents or legal guardians, and assent was obtained from children aged 12 years and older. The study protocol was approved by the local institutional ethics committee.

### Data collection

Demographic data (age, sex, family history of asthma or atopy) and clinical information (history of asthma exacerbations, current medications) were collected through structured interviews and review of medical records. Anthropometric measurements – including height (cm) and weight (kg) – were performed using standardized procedures. Body mass index (BMI) was calculated as weight (kg) divided by height squared (m^2^). BMI percentiles were classified according to the Centers for Disease Control and Prevention (CDC) growth charts: underweight (<5th percentile), normal weight (5th to <85th percentile), overweight (85th to <95th percentile), and obese (≥95th percentile).^20^

Asthma severity was assessed using clinical symptoms, medication use, and, when available, spirometry results (FEV1/FVC ratio), in accordance with the GINA guidelines.^1^ Severity was categorized as mild, moderate, or severe, based on symptom frequency, nocturnal symptoms, and lung function impairment.

### Laboratory measurements

Venous blood samples were collected from each participant after an overnight fast. Serum 25-hydroxy vitamin D [25(OH)D] levels were measured using chemiluminescent immunoassay (CLIA), with deficiency defined as 25(OH)D <30 ng/mL.^21^ Complete blood counts were performed using an automated hematology analyzer. Eosinophilia was defined as an absolute eosinophil count >500 cells/μL, consistent with previous pediatric studies.^22^ However, this relatively high cutoff may underestimate the true prevalence compared to lower thresholds (e.g., >300 cells/μL).

### Statistical analysis

Descriptive statistics were used to summarize demographic and clinical characteristics. Continuous variables were reported as mean ± standard deviation (SD) or median (interquartile range), depending on data distribution. Categorical variables were presented as frequencies and percentages. The chi-square test was used to compare proportions between groups, and one-way analysis of variance (ANOVA) or the Kruskal–Wallis test was used for continuous variables, as appropriate. Multivariate logistic regression was performed to assess the independent associations of obesity, vitamin D deficiency, and eosinophilia with asthma severity, adjusting for potential confounders such as age, sex, and family history. All statistical analyses were conducted using SPSS version 26 (IBM Corp., Armonk, NY, USA), with a two-tailed *p*-value <0.05 considered statistically significant.

## Results

A total of 177 children aged 6–18 years with physician-diagnosed asthma were included in the study. The mean age of participants was 10.4 ± 3.1 years, and the majority (69.5%) were male. Detailed demographic, clinical, and laboratory characteristics are presented in Table 1. Of the 177 children included, spirometry was successfully performed in 142 (80.2%), while 35 (19.8%) were diagnosed based on clinical criteria due to age-related or technical constraints (Table 1).

**Table 1:** Baseline characteristics of study participants.

Characteristic	Value (*n* = 177)
*Age (years), mean* ± *SD*	10.4 ± 3.1
*Male sex, n (%)*	123 (69.5)
*Height (cm), mean* ± *SD*	140.6 ± 18.2
*Weight (kg), mean* ± *SD*	39.6 ± 14.5

*Body mass index category, n (%)*	
Normal	113 (63.8)
Overweight	34 (19.2)
Obese	20 (11.3)

*Vitamin D deficiency, n (%)*	100 (56.5)
*Eosinophilia (>500/μL), n (%)*	18 (10.2)
*FEV1/FVC (%), mean* ± *SD*	80.4 ± 7.8

### Asthma severity distribution

Asthma severity was classified as mild in 74.6% (*n* = 132), moderate in 15.3% (*n* = 27), and severe in 10.2% (*n* = 18) of participants. The distribution of asthma severity by BMI category, vitamin D status, and eosinophilia is presented in Table 2.

**Table 2:** Distribution of asthma severity by body mass index, vitamin D status and eosinophilia.

Asthma severity	Mild (*n* = 132)	Moderate (*n* = 27)	Severe (*n* = 18)	*p*-Value
*BMI category*				
Normal	95 (72.0)	13 (48.1)	5 (27.8)	0.001
Overweight	25 (18.9)	7 (25.9)	2 (11.1)	
Obese	12 (9.1)	7 (25.9)	11 (61.1)	
*Vitamin D deficiency*	66 (50.0)	18 (66.7)	16 (88.9)	<0.001
*Eosinophilia*	6 (4.5)	3 (11.1)	9 (50.0)	<0.001

*Note*: Data are *n* (%) unless otherwise specified.

BMI: body mass index.

### Associations between risk factors and asthma severity

To further elucidate the relationships between risk factors and asthma severity, we examined the prevalence of overweight/obesity, vitamin D deficiency, and eosinophilia across severity groups (Table 3). Overweight/obesity and vitamin D deficiency were significantly more common in children with moderate or severe asthma compared to those with mild asthma. Eosinophilia was particularly prevalent in the severe asthma group.

**Table 3:** Prevalence of overweight/obesity, vitamin D deficiency and eosinophilia by asthma severity.

Risk factor	Mild (*n* = 132)	Moderate (*n* = 27)	Severe (*n* = 18)	*p*-Value
Overweight/obese	37 (28.0)	14 (51.9)	13 (72.2)	<0.001
Vitamin D deficiency	66 (50.0)	18 (66.7)	16 (88.9)	<0.001
Eosinophilia	6 (4.5)	3 (11.1)	9 (50.0)	<0.001

### Multivariate analysis

Multivariate logistic regression was performed to assess the independent associations of overweight/obesity, vitamin D deficiency, and eosinophilia with moderate–severe asthma (Table 4). After adjusting for age, sex, and family history of asthma, all 3 risk factors remained significantly associated with increased odds of moderate–severe asthma.

**Table 4:** Multivariate logistic regression for moderate–severe asthma.

Risk factor	Adjusted OR (95% CI)	*p*-Value
Overweight/obese	3.21 (1.87–5.51)	<0.001
Vitamin D deficiency	2.76 (1.45–5.25)	0.002
Eosinophilia	8.42 (3.24–21.89)	<0.001

### Conceptual model of risk factors and asthma severity

The conceptual model presents a multi-pathway model illustrating the complex interplay between various risk factors and asthma severity in children. The model is composed of patient characteristics, which include modifiable factors such as obesity and vitamin D status, as well as non-modifiable factors like age and sex; family history, which encompasses genetic predisposition, parental asthma, and atopic conditions; and environmental factors, which include exposures such as environmental tobacco smoke, indoor and outdoor allergens, air pollution, and infections.

These three clusters of risk factors interact with and influence biological pathways, which in turn include allergen sensitization, the process by which the immune system becomes sensitized to specific allergens, leading to allergic responses; allergic inflammation, characterized by the activation of immune cells (e.g., th2 lymphocytes, mast cells) and the release of cytokines and mediators; eosinophilic inflammation, a hallmark of allergic asthma, involving increased eosinophil counts and associated airway inflammation; pulmonary physiology, which encompasses lung growth, airway development, and lung function; rhinitis severity, which refers to the presence and severity of allergic rhinitis, commonly comorbid with asthma; and lung function, measured as FEV1/FVC and other spirometric parameters.

**Fig. 1:**
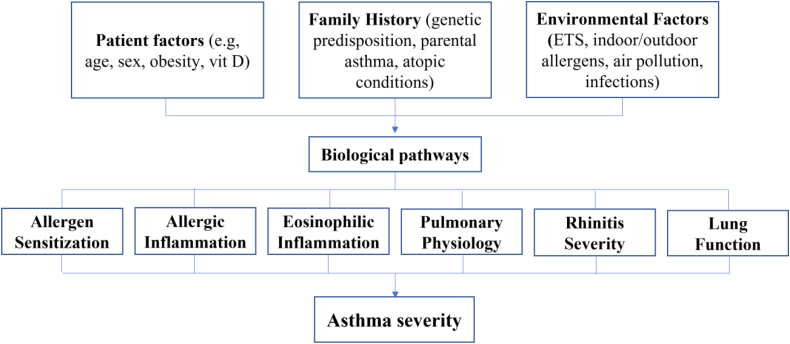
Conceptual model showing the independent associations of overweight/obesity, vitamin D deficiency, and eosinophilia with increased asthma severity in children.

These intermediate biological pathways ultimately converge to influence the outcome, asthma severity, which is the result of the complex interplay of the above factors, ranging from mild to severe disease (Fig. 1).

## Discussion

This study investigated the relationship between obesity, vitamin D deficiency, and eosinophilia with asthma severity among children aged 6–18 years in Arak, Iran. The findings indicate that all three factors were significantly associated with increased asthma severity, even after adjusting for potential confounders such as age, sex, and family history. These results support the growing recognition of modifiable risk factors in the pathophysiology and clinical progression of pediatric asthma.

The association between overweight/obesity and asthma severity observed in our study is consistent with previous research showing that higher BMI correlates with worse asthma control and lung function outcomes in children.^23^ Obesity may contribute to airway narrowing and systemic inflammation, reduce response to inhaled corticosteroids, and alter immune responses through mechanisms that are not yet fully understood.^24^ In our cohort, children categorized as overweight or obese were significantly more likely to experience moderate–severe asthma compared to their normal-weight peers.

Vitamin D deficiency, found in more than half of the participants, was also significantly associated with asthma severity. Vitamin D plays a critical immunomodulatory role and may influence the expression of inflammatory cytokines involved in asthma pathogenesis.^11^ Prior studies have reported an inverse association between serum 25(OH)D levels and asthma severity, with lower levels linked to increased airway hyperresponsiveness, frequent exacerbations, and steroid resistance.^25^ Our findings are in line with meta-analyses that suggest children with asthma may benefit from maintaining adequate vitamin D levels, although randomized controlled trials have yielded mixed results.^13^, ^26^

Eosinophilia, which was more prevalent among children with severe asthma, emerged as the strongest independent predictor of asthma severity in this study. Elevated eosinophil counts reflect eosinophilic inflammation, a key feature of allergic asthma that contributes to airway remodeling, exacerbations, and poor symptom control.^15^ In line with previous research, our data indicate that eosinophilic inflammation remains an important biomarker for asthma severity, and its presence should prompt consideration of more aggressive anti-inflammatory therapy, including the use of biologics in severe cases.^14^, ^27^

The model presented in this study features several advances over traditional conceptual models of asthma. It explicitly clusters risk factors into patient, family, and environmental domains, consistent with current consensus models in pediatric asthma.^28^ This clustering highlights the multidimensional nature of asthma risk and aligns with recommendations from expert panels that advocate for comprehensive, multi-domain approaches to asthma conceptualization and prevention. By mapping out specific biological pathways, such as allergen sensitization, allergic and eosinophilic inflammation, pulmonary physiology, and rhinitis severity, the model clarifies how upstream risk factors mechanistically contribute to asthma severity. This level of detail is supported by current understanding of asthma pathogenesis, which emphasizes the roles of Th2-mediated inflammation, eosinophilia, and airway remodeling.^29^

It also demonstrates the interconnectedness of risk factors and pathways, which is critical for understanding the multifactorial nature of asthma. This approach is in line with recent calls for models that can capture the interplay among codependent risk factors and multiple domains affecting asthma diagnosis and severity.^28^ By illustrating the convergence of risk factors and pathways on asthma severity, the model supports the development of targeted, multi-component interventions. This is particularly relevant for public health and clinical decision-making, as highlighted in recent studies that recommend microsimulation and open-population modeling to evaluate the impact of interventions across diverse risk domains.

This study adds value by examining the combined effect of 3 modifiable or clinically observable factors – BMI, vitamin D status, and eosinophilia – on asthma severity, rather than treating each as an isolated variable. The synergistic effect of these risk factors suggests that a multi-pronged approach may be most effective in asthma prevention and control strategies.

### Limitations

Several limitations should be acknowledged. First, the cross-sectional design precludes causal inference. Second, the study was conducted in a single referral center, which may limit generalizability. Third, although 80% of participants underwent spirometry, a subset of younger children was diagnosed based only on clinical criteria, which could introduce diagnostic variability. Fourth, our sample was characterized by a predominance of males (69.5%) and a relatively small proportion of adolescents (13–18 years), raising the possibility of inclusion bias. Fifth, most cases were classified as mild asthma (74.6%), likely reflecting referral and follow-up patterns in our center, which could limit extrapolation to more severe populations. Finally, the cutoff used to define eosinophilia (>500 cells/μL) may have underestimated its prevalence compared with more commonly used thresholds (e.g., >300 cells/μL).

## Conclusion

This study found that overweight/obesity, vitamin D deficiency, and eosinophilia are independently and significantly associated with greater asthma severity in children. These findings emphasize the importance of identifying and managing modifiable risk factors in pediatric asthma care. An integrated, multi-factor approach – including nutritional assessment, vitamin D monitoring, and eosinophil evaluation – could improve asthma control and reduce disease burden. Future research should focus on confirming these associations through prospective studies and guiding targeted, population-specific interventions.

## Ethics approval and informed consent

This study was approved by the Ethics Committee of Arak University of Medical Sciences (Approval Code: IR.ARAKMU.REC.1403.215). Written informed consent was obtained from parents or legal guardians of all participants, and assent was obtained from children aged 12 years and older. All procedures were conducted in accordance with the Declaration of Helsinki.

## Declaration of generative AI and AI-assisted technologies in the writing process

During the preparation of this work, the authors used *ChatGPT* (*OpenAI*) to improve the language and readability of the manuscript. After using this tool, the authors reviewed and edited the content as needed and take full responsibility for the content of the publication.

## Funding

This research was supported by the 10.13039/501100007113Arak University of Medical Sciences, Arak, Iran.

## Authors’ contributions

MR contributed to conceptualization, data collection, and manuscript drafting. MM was responsible for data analysis, interpretation, and literature review. SA provided methodological supervision, manuscript editing, and critical revision. JN was responsible for study design, overall supervision, final manuscript approval, and corresponding author duties.

## Conflict of interest

The authors declare that there are no conflicts of interest regarding the publication of this article.
